# Anesthetic management of a child with Kagami-Ogata syndrome complicated with marked tracheal deviation: a case report

**DOI:** 10.1186/s40981-018-0199-5

**Published:** 2018-08-31

**Authors:** Kazuaki Yamagata, Atsushi Kawamura, Satomi Kasai, Mai Akazawa, Michiru Takeda, Kazuya Tachibana

**Affiliations:** 1 Department of Dental Anesthesiology, Field of Oral and Maxillofacial Rehabilitation, Kagoshima University Graduate School of Medical and Dental Sciences, 8-35-1 Sakuragaoka, Kagoshima-shi, Kagoshima 890-8544 Japan; 2 Department of Anesthesiology, Osaka Women’s and Children’s Hospital, 840 Murodo-cho, Izumi-shi, Osaka 594-1101 Japan; 3 Department of Anesthesiology, Osaka Minami Medical Center, 2-1 Kidohigashi-machi, Kawachinagano-shi, Osaka 586-8521 Japan; 4 Department of Anesthesiology, Shiga University of Medical Science, Seta Tsukinowa-cho, Otsu-shi, Shiga 520-2134 Japan

**Keywords:** Kagami-Ogata syndrome, Paternal uniparental disomy 14, Bell-shaped small thorax, Coat-hanger sign, Respiratory distress, Tracheal deviation

## Abstract

**Background:**

Kagami-Ogata syndrome (KOS) is a rare congenital imprinting disorder. The problems related to the anesthetic management of patients with KOS are respiratory distress and difficult endotracheal intubation.

**Case presentation:**

A 2-year-old male was scheduled to undergo orchiopexy for bilateral cryptorchidism. Although he had a history of severe respiratory distress immediately after birth, his preoperative respiratory condition was stable. He also had marked tracheal deviation. General anesthesia was induced with nitrous oxide and sevoflurane in oxygen. A laryngeal mask airway (LMA) was inserted following rocuronium administration. Anesthesia was maintained with sevoflurane and simultaneous caudal anesthesia. His postoperative course was uneventful.

**Conclusions:**

Patients with KOS should preferably undergo elective surgery only after infancy because their respiratory status is more stable as they grow older. Thorough preoperative evaluation of the respiratory tract is important even in KOS patients with a stable respiratory condition.

## Background

Kagami-Ogata syndrome (KOS) is a congenital disease caused by abnormal expression of the imprinted genes in chromosome 14q32 [1]. It was first reported in 1991 as a phenotype of paternal uniparental disomy 14 [2]. In 2015, Kagami et al. defined KOS by summarizing its onset mechanisms and diagnostic criteria [3]. KOS is characterized by craniofacial dysmorphism and thoracic abnormalities. Craniofacial dysmorphism includes a depressed nasal bridge, full cheeks, protruding philtrum, micrognathia, and short-webbed neck. Thoracic abnormalities include a small bell-shaped thorax in infancy and coat-hanger appearance of the ribs from infancy through childhood. Although there are various clinical symptoms, such as amniotic fluid excess, dysphagia, and respiratory distress, the most problematic symptom is respiratory distress due to thoracic abnormalities. KOS is associated with a poor prognosis because of severe respiratory distress [4].

Only approximately 50 patients with KOS have been identified in the literature [1], and its anesthetic management has not been previously described. In this report, we present the successful anesthetic management of a child with KOS complicated with tracheal deviation.

## Case presentation

The patient was a 2-year-old male (weight 11.5 kg; height 83.5 cm) who had been delivered vaginally at 31 weeks of gestation with a birth weight of 2258 g. He was suspected to have KOS due to excessive amniotic fluid in the fetal period and his physical characteristics after birth. The diagnosis was confirmed by genetic analysis. Immediately after birth, he had developed severe respiratory distress and had required mechanical ventilation for 20 days; he had been managed in the neonatal intensive care unit for 4 months. Even after withdrawal of mechanical ventilation, oxygen administration had been required for up to 9 months of age, although his respiratory condition thereafter stabilized and continued to be stable in the preoperative period. He had no previous surgical history.

**Fig. 1:**
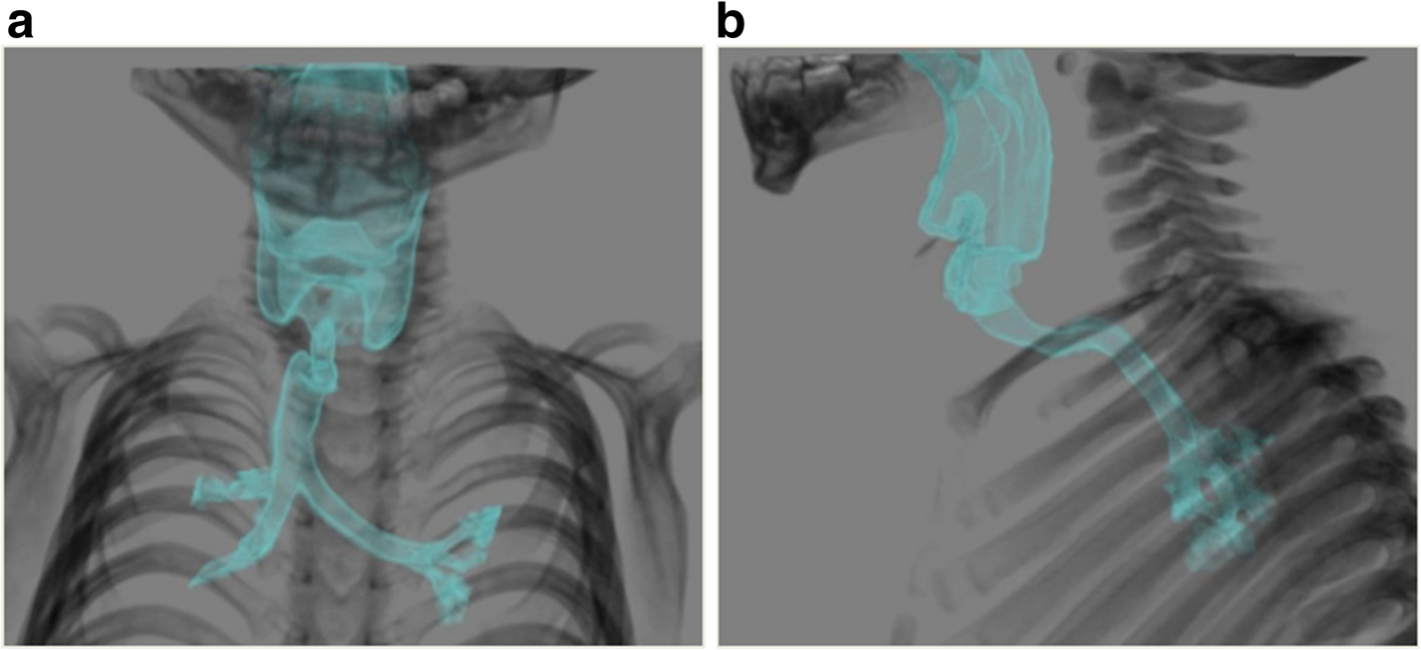
Computed tomography before operation. **a** Frontal view. **b** Lateral view. The trachea was severely deviated at an angle of 90 ° from the ventral to dorsal side

At the age of 2 years, he was scheduled to undergo orchiopexy for bilateral cryptorchidism. Preoperative computed tomography examination indicated deviation of the trachea, with flexion from the ventral to the dorsal side (Fig. 1). There were no abnormalities in other examination findings.

Our plan A was to insert LMA after slow induction and plan B was to attempt fiberoptic intubation (FOI) with endoscopy mask. Endoscopy mask allows for continued positive pressure mask ventilation while providing a site for FOI. The patient was given 0.5 mg/kg midazolam orally as premedication 30 min before the operation. Standard monitoring was applied. General anesthesia was induced via a face mask with sevoflurane and nitrous oxide in 30% oxygen. After confirming that mask ventilation was possible, the sevoflurane concentration was gradually increased. After disappearance of spontaneous respiration, an intravenous catheter was inserted and 0.9 mg/kg rocuronium was administered. Subsequently, LMA (size 2.0) was uneventfully inserted. He was then positioned in the lateral position, and caudal anesthesia was administered using ropivacaine (0.25%, 10 mL). Anesthesia was maintained with 3% sevoflurane, air (3 L/min), and oxygen (1 L/min). He was ventilated using conventional pressure-controlled ventilation (PCV) with a positive end-expiratory pressure of 5 cmH2O, peak inspiratory pressure of 15 cmH2O, frequency of 20 breaths/min, and inspiratory time of 0.8 s. Expiratory tidal volume was approximately 110 mL. SpO2 and end-tidal CO2 were maintained at 98–99% and approximately 35 mmHg, respectively. At the end of surgery, muscle relaxation was antagonized by sugammadex sodium (4 mg/kg). After confirmation of spontaneous respiration, the LMA was removed. Anesthesia time was 161 min. Since there were no adverse respiratory events, the patient was transferred to the general ward. His subsequent recovery was uneventful, and he was discharged from the hospital the next day.

## Discussion

The issues involved in the anesthetic management of patients with KOS are respiratory distress due to thoracic abnormalities and difficult endotracheal intubation due to craniofacial dysmorphism.

**Fig. 2:**
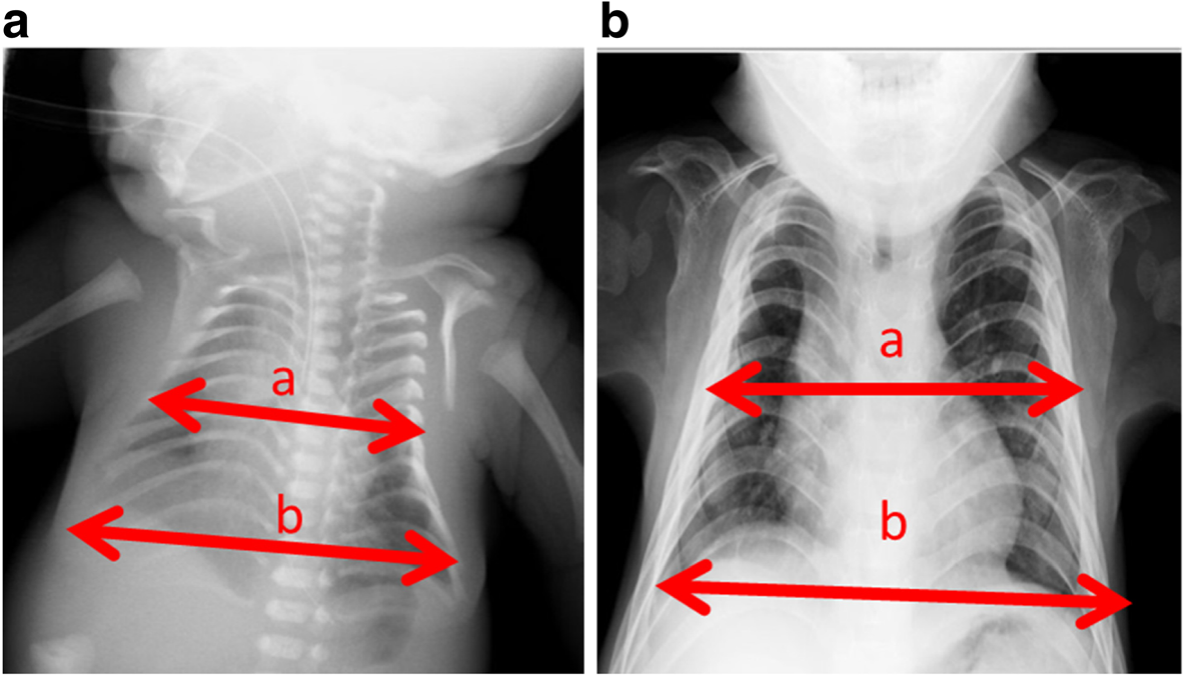
Examples of M/W ratios (the ratio of the mid to widest thoracic diameter.) M/W ratio = *a*/*b* × 100. **a** At birth. The M/W ratio was 67% (normal range 82–89%). **b** At 1 year 10 months (before operation). The M/W ratio was 79% (normal range 83–98%). The M/W ratio tended to improve with age

Respiratory distress in patients with KOS develops immediately after birth, requiring intubation and mechanical ventilation in most cases. The duration of mechanical ventilation is variable, with a median period of 1 month, and is apparently unrelated to gestational age [2]. The mechanism of this respiratory distress has not yet been clarified, although there are several possible explanations. Structural thoracic abnormalities are presumed to be part of the cause, leading to reduced lung volume, functional residual capacity (FRC), and low thoracic compliance, generally resulting in restrictive respiratory disease. The small thorax is also seen in patients with Jeune syndrome, campomelic dysplasia, thanatophoric dysplasia, and achondrogenesis [5]. Jeune syndrome is a rare condition [6] that primarily affects the bones. Common signs and symptoms include a small thorax and short ribs which restrict the growth and expansion of the lungs, often causing life-threatening breathing difficulties [7]. In many cases, the cause of Jeune syndrome is unknown. Changes in several different genes have been identified in some families with the condition. However, the small bell-shaped thorax and coat-hanger appearance of the ribs are specific to patients with KOS [2]. A previous report evaluated the ratio of the mid to widest thoracic diameter (M/W ratio) in patients with small bell-shaped thoraxes, and the angle between the sixth posterior rib and the horizontal axis (coat hanger angle, CHA) for the coat-hanger appearance [8]. According to the literature, the CHA in patients with KOS remains above the normal range for age-matched control children, while the M/W ratio, although below the normal range in infancy, becomes within the normal range after infancy. Furthermore, as the M/W ratio improves with growth, respiratory distress becomes less severe [2]. Our patient developed severe respiratory distress immediately after birth, which required mechanical ventilation with tracheal intubation. However, his preoperative respiratory condition was stable and he did not require a high driving pressure during the operation. This is because the M/W ratio of his small bell-shaped thorax tended to improve with growth, from 67% at birth to 79% before surgery (Fig. 2). This indicates that the M/W ratio could aid effective evaluation of the preoperative respiratory condition in patients with KOS.

Tracheal intubation in patients with KOS is predicted to be difficult because of the presence of certain craniofacial anomalies, such as micrognathia and a short-webbed neck. Additionally, our case was complicated by marked tracheal deviation. His trachea was extremely deviated at an angle of 90 ° from the ventral to dorsal side. Since this tracheal deviation was not recognized on his chest X-ray at birth, it was thought to have probably developed during the growth process. However, it is unclear whether this tracheal deviation is specific to patients with KOS, because there are no previous reports of tracheal deviation in patients with KOS. The tracheal deviation could be caused by various factors, including a thyroid goiter, a tumor in the neck and thorax, trauma, lung collapse, or spinal deformities [9]. Whatever the cause may be, in most previous reports, the authors tried to advance the tracheal tube below the deviated area and were usually successful [9]. However, there are a few reports of failure to intubate below the deviated area. Davies reported that it was impossible to intubate a patient with a combination of tracheal deviation and tracheal diverticulum [10]. In that case, an LMA was used to secure the airway. Kim et al. reported that the tracheal tube could not be advanced more than 3 cm beyond the vocal cords in a patient with tracheal deviation due to kyphoscoliosis [7].

In our case, we decided to use LMA as the airway device under consideration of his preoperative respiratory condition, tracheal deviation, and planned surgical procedure. Subsequent anesthetic management was performed successfully.

## Conclusions

The issues related to the anesthetic management of patients with KOS are respiratory distress due to thoracic abnormalities and difficult endotracheal intubation due to craniofacial dysmorphism. Detailed preoperative evaluation of the respiratory tract in KOS patients is necessary even in those with a stable respiratory condition.

## Abbreviations

CHA: Coat hanger angle
KOS: Kagami-Ogata syndrome
LMA: Laryngeal mask airway
M/W ratio: Ratio of the mid to widest thoracic diameter
PCV: Pressure-controlled ventilation
